# Different packing motifs mediated by hydrogen bonds in the hydro­chloride salts of two pyridoxal *N*-acyl­hydrazone derivatives

**DOI:** 10.1107/S2056989025007856

**Published:** 2025-09-09

**Authors:** Thais C. M. Nogueira, Marcus V. N. deSouza, James L. Wardell, William T. A. Harrison

**Affiliations:** a Fundação Oswaldo Cruz, Instituto de Tecnologia em Fármacos-Far, Manguinhos, 21041-250, Rio de Janeiro, RJ, Brazil; b Department of Chemistry, University of Aberdeen, Meston Walk, Aberdeen AB24 3UE, Scotland, United Kingdom; Texas A & M University, USA

**Keywords:** crystal structure, pyridoxal ring, hydro­chloride salt, hydrogen bonding

## Abstract

The crystal structures of two hydro­chloride salts of pyridoxal–*N*-acyl­hydrazone–Q (Q = heterocyclic aromatic ring) derivatives, *viz*. (*E*)-3-hy­droxy-5-(hy­droxy­meth­yl)-2-methyl-4-{[(pyridin-4-ylformamido)­imino]­meth­yl}pyridin-1-ium chloride dihydrate, C_14_H_15_N_4_O_3_^+^·Cl^−^·2H_2_O, (**I**), and (*E*)-3-hy­droxy-5-(hy­droxy­meth­yl)-2-methyl-4-{[(pyrimidin-2-ylformamido)­imino]­meth­yl}pyridin-1-ium chloride dihydrate, C_13_H_14_N_5_O_3_^+^·Cl^−^·2H_2_O, (**II**) are described. The cations, which are protonated at the pyridine N atom of the pyridoxal ring, have similar overall conformations: the dihedral angles between the pyridoxal ring and the terminal aromatic ring are 12.63 (12) and 6.11 (15)° for (**I**) and (**II**), respectively. Each cation features an intra­molecular O—H⋯N hydrogen bond, which closes an *S*(6) ring, but a difference arises in the conformation of the C—C—C—O fragment terminated by the the ring carbon atom bonded to the side chain and the O atom of the hy­droxy­methyl group: *gauche* for (**I**) and *anti* for (**II**). The extended structures of (**I**) and (**II**) feature numerous strong (N—H and O—H donors) and weak (C—H donor) hydrogen bonds. In (**I**), the NH_p_ (pyridine) grouping links to the terminal N atom of the pendant unprotonated pyridine ring of an adjacent cation to generate [010] chains, whereas the NH_h_ (hydrazide) and OH_hm_ (hy­droxy­meth­yl) moieties link to chloride ion acceptors. In (**II**), the NH_p_ and OH_hm_ groupings bond to chloride anions whereas NH_h_ bonds to a water mol­ecule. Hydrogen-bonded chains of water mol­ecules occur in (**I**) and centrosymmetric tetra­mers in (**II**). The Hirshfeld surfaces of (**I**) and (**II**) are computed and the structures of related compounds are briefly compared.

## Abstract 2

The closely related title compounds show quite different hydrogen-bonding motifs from the same donor atoms in the cations.

## 1. Chemical context

Vitamin B6 is a water-soluble vitamin that is naturally present in many foods, added to others, and available as a dietary supplement. It is crucial for various bodily functions, including energy metabolism, nerve function, brain development, and immune support and numerous reviews have been published (*e.g*., Stach *et al.*, 2021 ▸; Muhamad *et al.*, 2023 ▸; Santos *et al.*, 2023 ▸). Vitamin B6 is the generic name for a group of six compounds (vitamers), which can readily be inter­converted *via* biochemical pathways, namely pyridoxine (with a hy­droxy­methyl group *trans* to the pyridine N atom), pyridoxal (an aldehyde) and pyridoxamine (an amine) and their respective 5′-phosphate esters (see Fig. 1 of Stach *et al.*, 2021 ▸). Pyridoxal 5′ and pyridoxamine 5′ phosphates are the active coenzyme forms of vitamin B6. Vitamin B6–metal complexes (Casas *et al.*, 2012 ▸; Gupta, 2022 ▸) and chemical modifications of the vitamers have been widely studied for their biological activities (Pawar *et al.*, 2023 ▸). In keeping with the general finding that hydrazonyl and acyl­hydrazonyl compounds have potentially useful biological activities (Socea *et al.*, 2022 ▸), various derivatives of the B6 vitamers have been shown to have bio-activities, and have been studied for their iron chelating (Bartolić *et al.*, 2024 ▸) and anti-tumour activities (Chen *et al.*, 2019 ▸) and, by some of us, for their anti-microbial and anti-tuberculosis properties (Nogueira *et al.*, 2019 ▸).
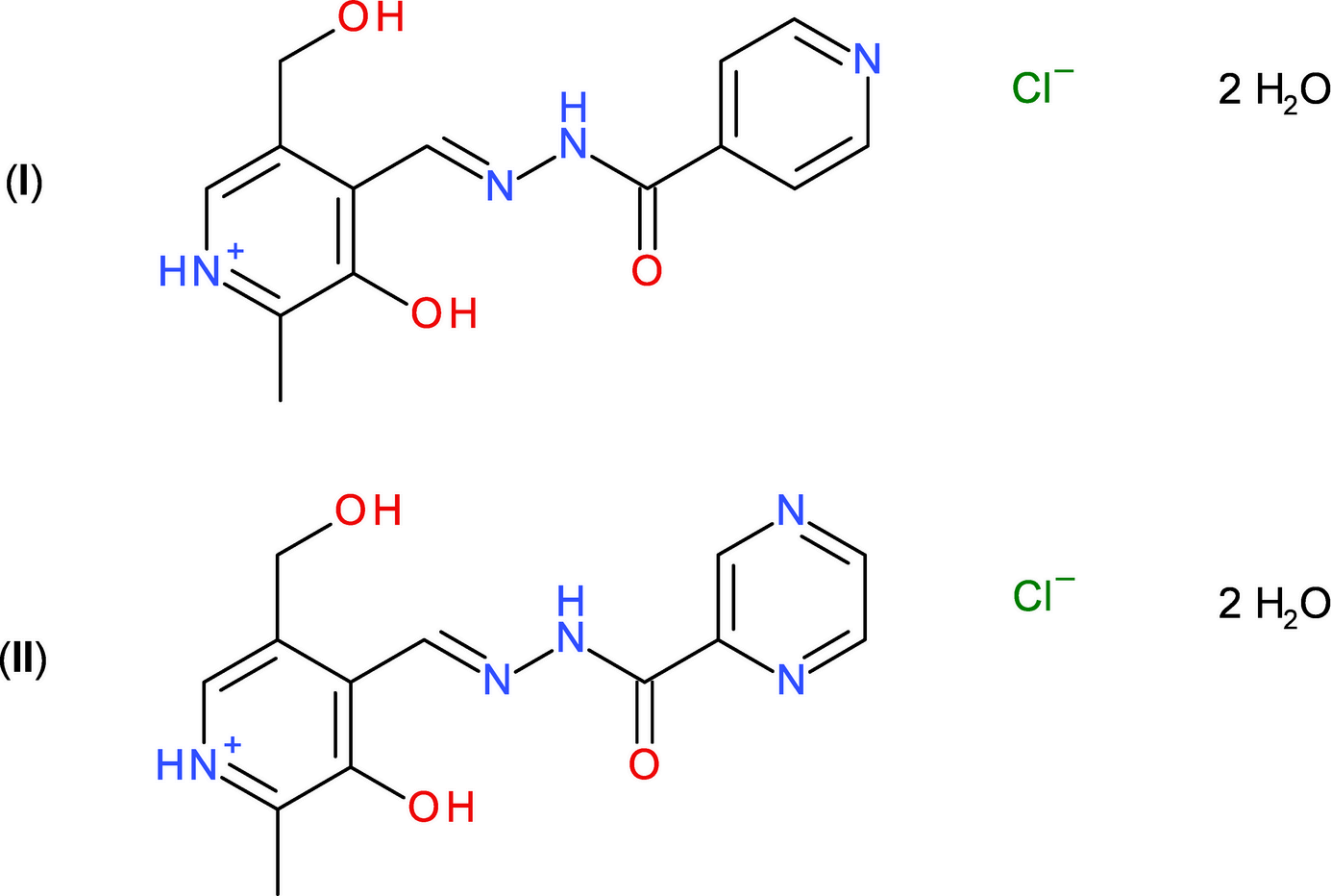


In continuation of our earlier work (Nogueira *et al.*, 2019 ▸), we now describe the crystal structures and Hirshfeld surfaces of (*E*)-3-hy­droxy-5-(hy­droxy­meth­yl)-2-methyl-4-{[(pyridin-4-ylformamido)­imino]­meth­yl}pyridin-1-ium chloride dihydrate, C_14_H_15_N_4_O_3_^+^·Cl^−^·2H_2_O, (**I**) and (*E*)-3-hy­droxy-5-(hy­droxy­meth­yl)-2-methyl-4-{[(pyrimidin-2-ylformamido)­imino]­meth­yl}pyridin-1-ium chloride dihydrate, C_13_H_14_N_5_O_3_^+^·Cl^−^·2H_2_O, (**II**).

## 2. Structural commentary

The crystal structures of (**I**) and (**II**) confirm the atomic connectivities of the (stated) neutral mol­ecules described in the previous study (Nogueira *et al.*, 2019 ▸): here, each compound crystallizes as a hydro­chloride salt protonated at the pyridine N atom of the pyridoxal ring, accompanied by two water mol­ecules of crystallization.

**Figure 1:**
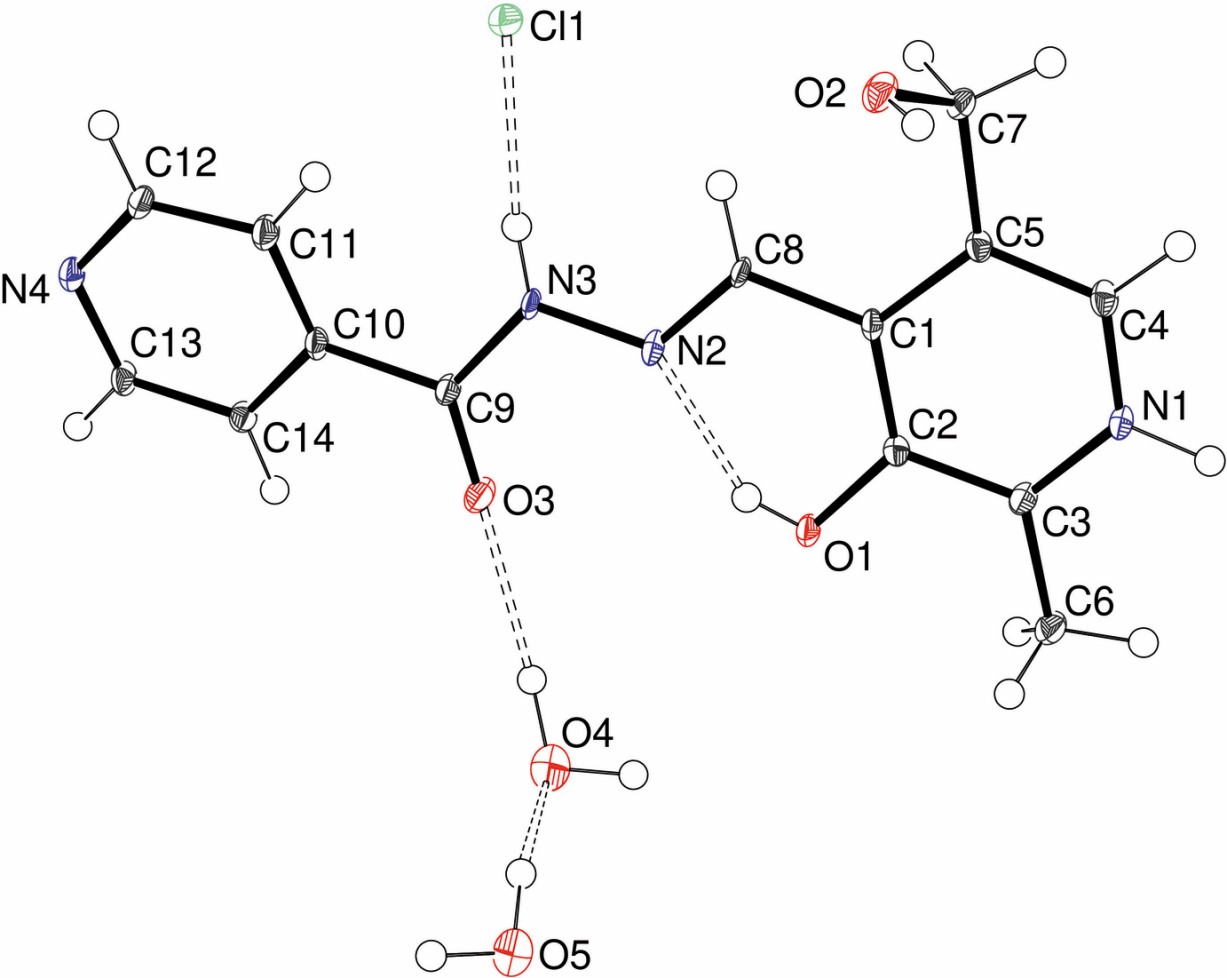
The mol­ecular structure of (**I**) showing 50% displacement ellipsoids. The hydrogen bonds are indicated by double-dashed lines.

Compound (**I**) crystallizes in the monoclinic space group *Ia*, with one cation, one chloride counter ion and two water mol­ecules of crystallization in the asymmetric unit (Fig. 1 ▸). In the C_14_H_15_N_4_O_3_^+^ cation, the C8=N2 double bond is in an *E* configuration, with a C1—C8—N2—N3 torsion angle of −179.1 (2)°. The C8—N2—N3—C9 torsion angle is −179.8 (2)° and oxygen atoms O1 and O3 lie to the same side of the mol­ecule. The C1—C5—C7—O2 torsion angle associated with the hy­droxy­methyl group is 61.9 (3)°, *i.e*., O2 is *gauche* with respect to C1, and the dihedral angle between the pyridoxal C1–C5/N1 pyridine ring and the pendant C10–C14/N4 pyridine ring is 12.63 (12)°, with the most significant twist occurring about the C9—C10 bond [N3—C9—C10—C11 = −13.5 (3)°]. The N2—N3 bond length of 1.367 (3) Å is significantly shorter than a typical N—N single bond (∼1.44 Å), which suggests substantial delocalization of electrons (*i.e*., resonance forms) between the C8=N2 double bond and C8=O3 carbonyl group of the carbohydrazide grouping as observed previously for related compounds (Cardoso *et al.*, 2016 ▸). An intra­molecular O1—H1*O*⋯N2 hydrogen bond (Table 1 ▸) closes an *S*(6) loop. Otherwise, the bond lengths and angles in (**I**) may be regarded as unexceptional.

**Figure 2:**
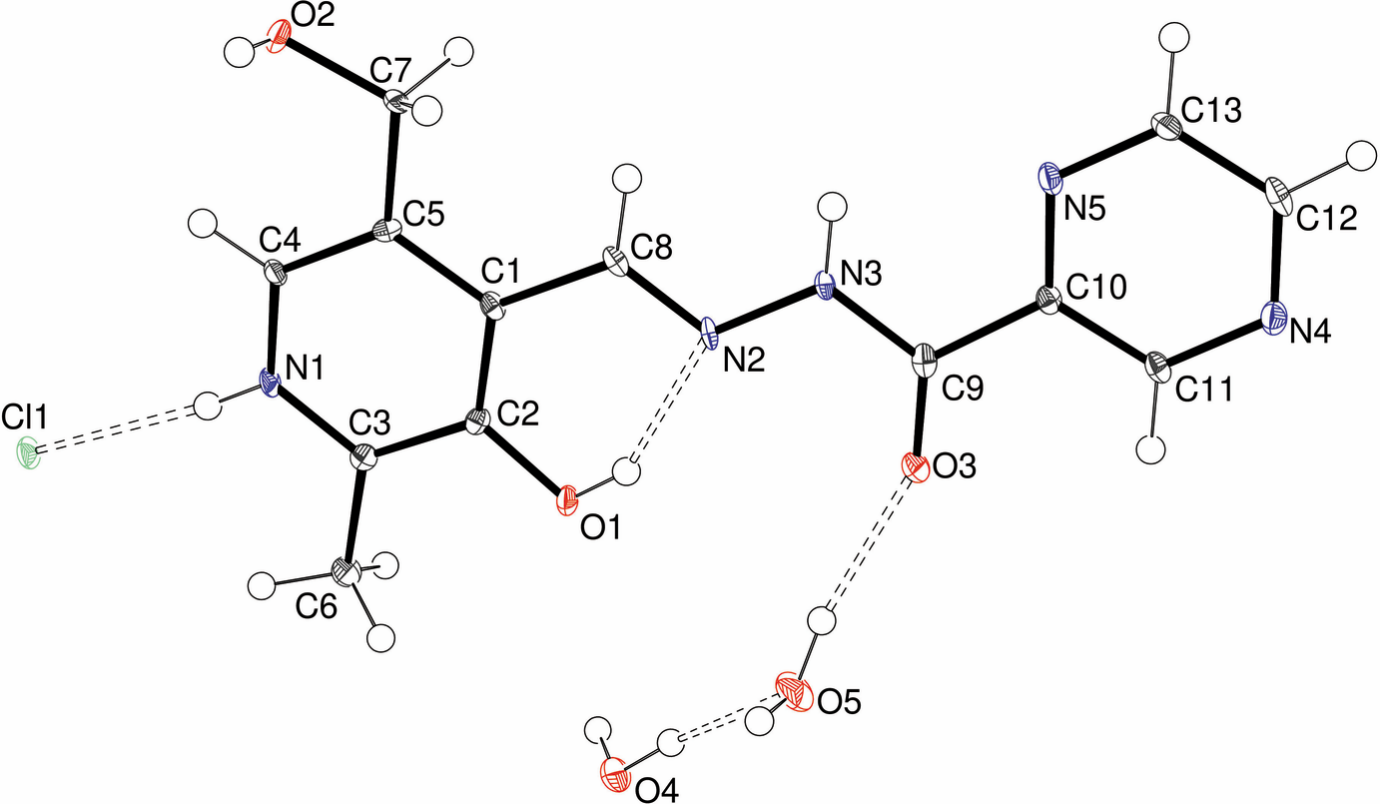
The mol­ecular structure of (**II**) showing 50% displacement ellipsoids. The hydrogen bonds are indicated by double-dashed lines.

In compound (**II**), the pyrimidine analogue of (**I**), there are one cation, one chloride anion and two water mol­ecules of crystallization in the asymmetric unit (Fig. 2 ▸) in the triclinic space group *P*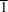. In the C_13_H_14_N_5_O_3_^+^ cation, the C8=N2 double bond is in an *E* configuration, with C1—C8—N2—N3 = 178.2 (3)° and C8—N2—N3—C9 = 179.5 (3)°. The C1—C5—C7—O2 torsion angle of 178.2 (3)° indicates an *anti* conformation for O2 and the dihedral angle between the C1–C5/N1 pyridine ring and C10–C13/N4/N5 pyrimidine ring is 6.11 (15)°, with the largest twist occurring about the C1—C8 bond [C2—C1—C8—N2 = −3.3 (5)°]. The N2—N3 bond length is 1.373 (4) Å and, as in (**I**), an intra­molecular O—H⋯N hydrogen bond (Table 2 ▸) occurs.

## 3. Supra­molecular features

Geometrical data for the directional inter­molecular inter­actions in (**I**) and (**II**) are listed in Tables 1 ▸ and 2 ▸, respectively. As well as the intra­molecular links involving O1—H1*O*⋯N2 noted above, the cations in these structures have the ability to form three strong inter­molecular hydrogen bonds, from the pyridine N1—H1*N*, the hydrazide N3—H2*N* and the hy­droxy­methyl O2—H2*O* groupings and these form quite different arrangements in these two structures.

**Figure 3:**
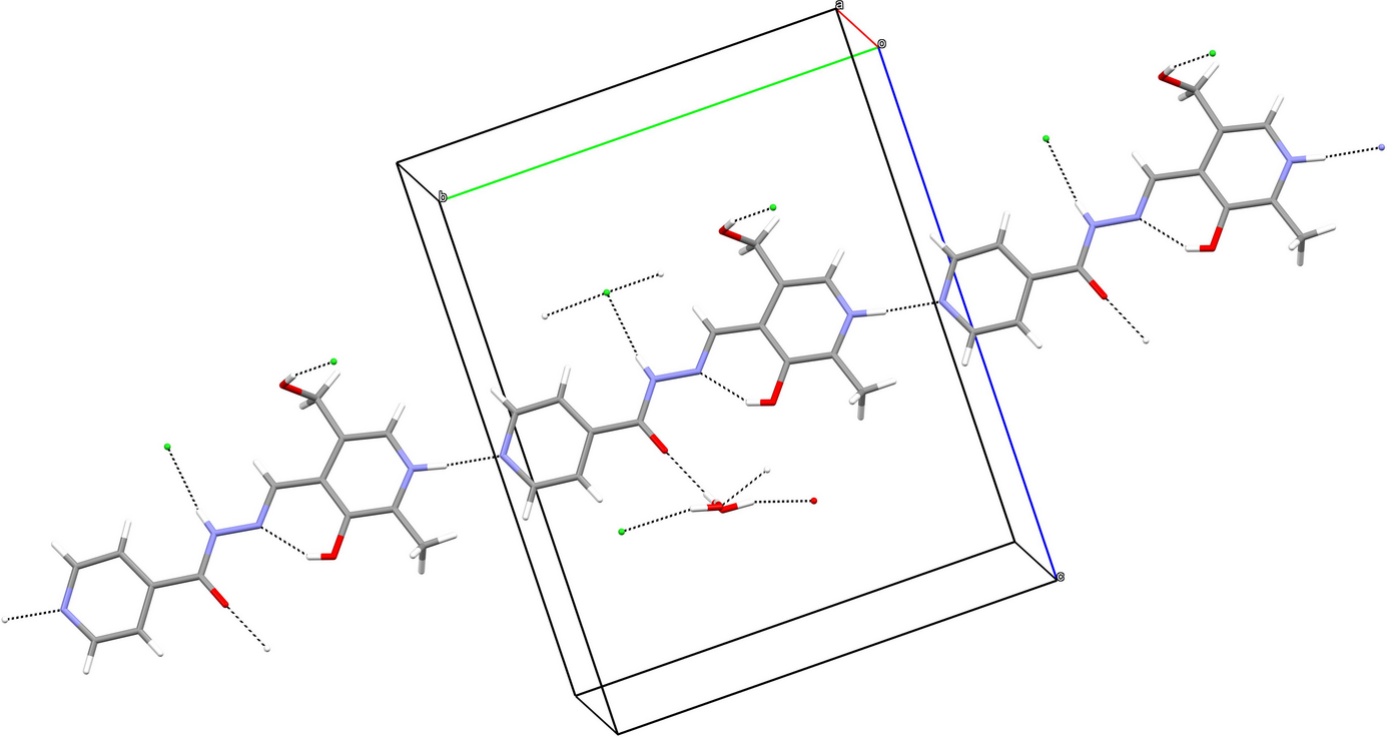
Fragment of the crystal structure of (**I**) showing hydrogen bonds as dashed lines.

**Figure 4:**
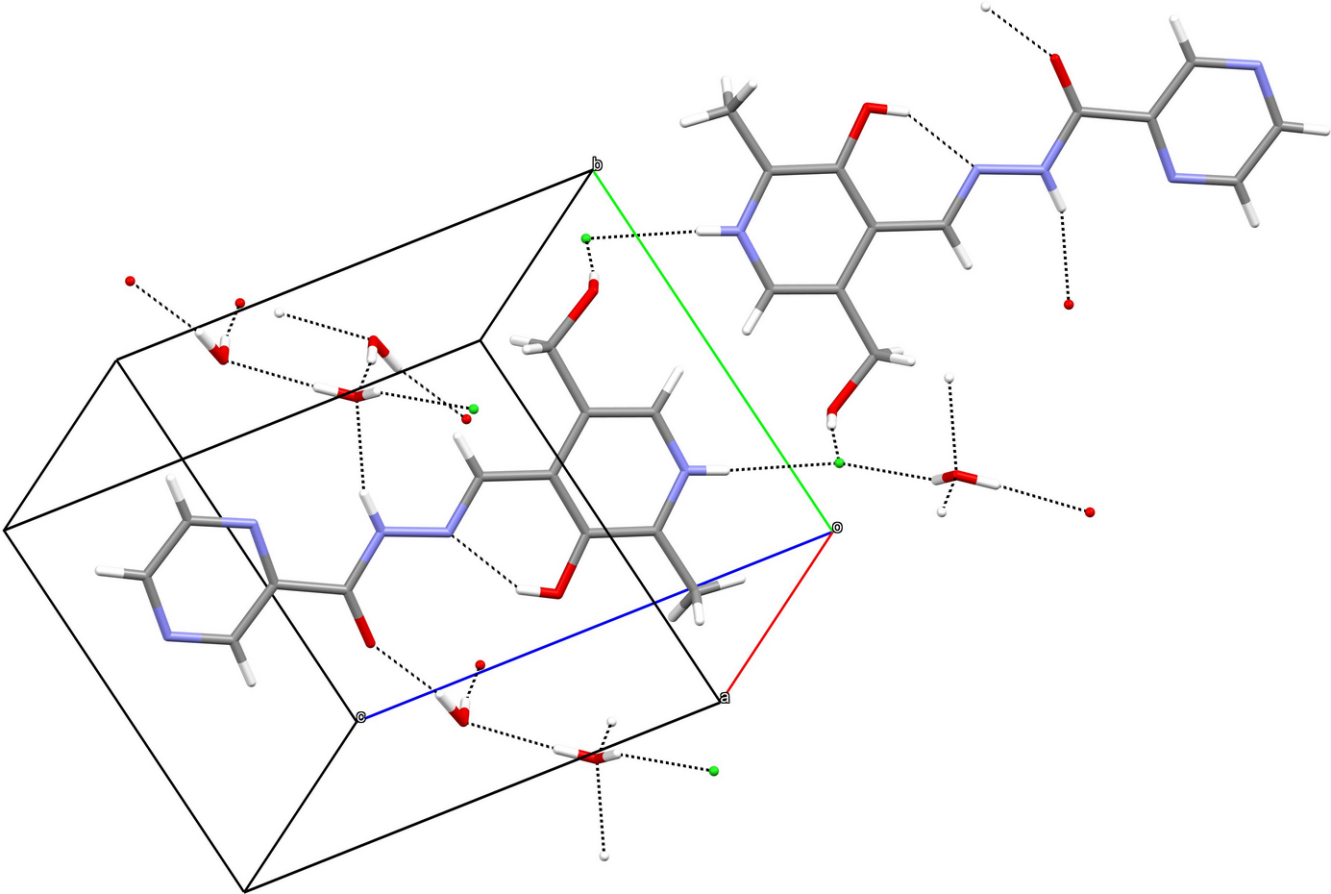
Fragment of the crystal structure of (**II**) showing hydrogen bonds as dashed lines.

**Figure 5:**
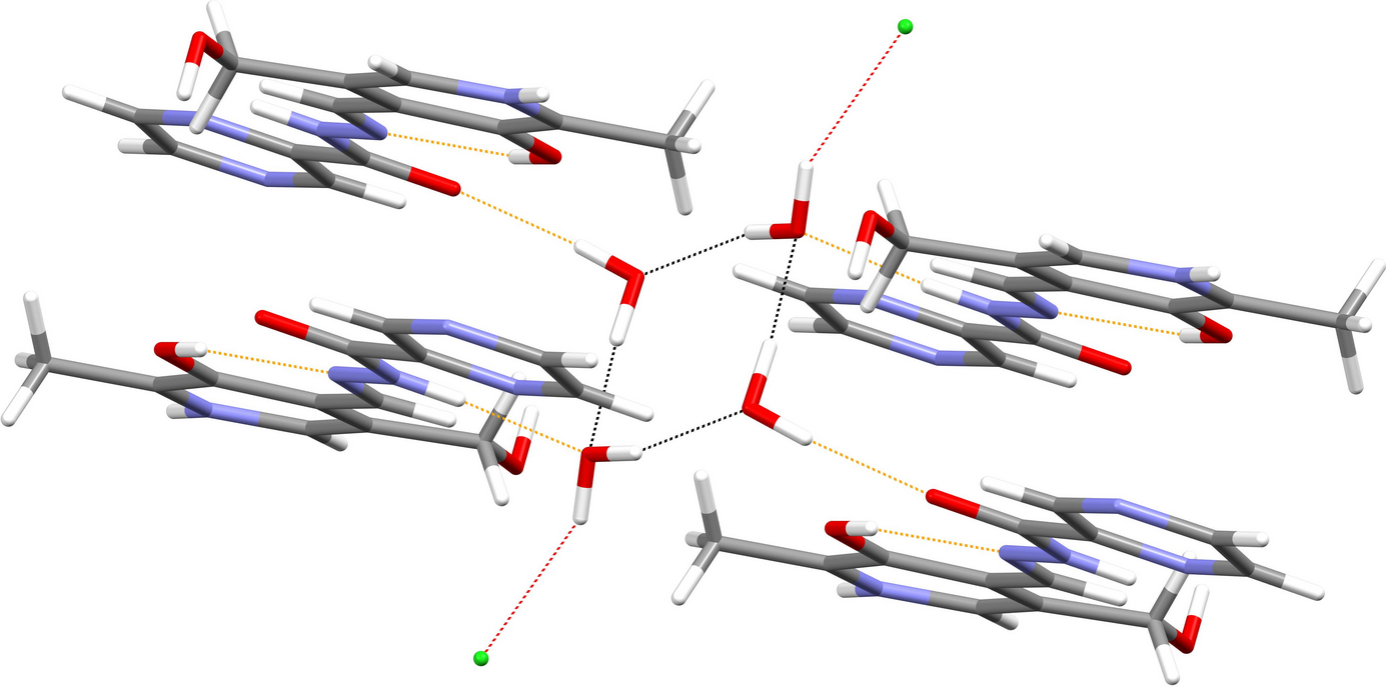
Cyclic tetra­mer of hydrogen-bonded water mol­ecules in the extended structure of (**II**). The hydrogen bonds associated with the tetra­mer are rendered in black and the other hydrogen bonds are coloured orange.

In (**I**), the N1—H1*N* grouping links to the terminal N4 atom of the pendant pyridine ring of an adjacent cation displaced by translation in the *b*-axis direction, to generate [010] chains of cations, whereas N3—H2*N* and O2—H2*O* link to nearby chloride ions (Fig. 3 ▸). Conversely, in (**II**), the N1—H1*n* and O2—H2*O* moieties make links to chloride anions whereas N3—H2*N* bonds to a water mol­ecule (Fig. 4 ▸). The O4 water mol­ecule in (**I**) forms O—H⋯O hydrogen bonds to the ketone O atom of the cation and the other water mol­ecule, whilst the O5 water mol­ecule forms a hydrogen bond to the other water mol­ecule (thereby forming infinite [100] chains of water mol­ecules) and one link to a chloride ion. In (**II**), the water mol­ecules form the same local pattern of hydrogen bonds as in (**I**), but here a completely different supra­molecular *motif* of centrosymmetric tetra­mers of hydrogen-bonded water mol­ecules arises (Fig. 5 ▸). These two structures also feature various weak C—H⋯*X* (*X* = N, O, Cl) inter­actions as listed in the hydrogen-bond tables; it may be mentioned that there are no fewer than nine of these bonds in (**I**) compared to just four in (**II**). The shortest aromatic ring centroid–centroid separations in these structures are π_q_–π_q_ = 3.8543 (14) Å (slippage = 2.062 Å) for (**I**) and π_p_–π_q_ = 3.4724 (18) Å (slippage = 1.047 Å) for (**II**) where π_p_ and π_q_ are the centroids of the pyridoxal ring and pendant aromatic ring, respectively.

In order to gain more insight into these different packing motifs, the Hirshfeld surfaces and fingerprint plots for (**I**) and (**II**) were calculated using *CrystalExplorer* (Spackman *et al.*, 2021 ▸) following the protocol of Tan *et al.* (2019 ▸). The Hirshfeld surfaces (see supplementary materials) show the expected red spots (close contacts) in the vicinities of the various donor and acceptor atoms of the respective cations noted in the previous paragraph. The fingerprint plots decomposed into the different percentage contact types (Table 3 ▸) show that the most important contributions are H⋯H, O⋯H/H⋯O and C⋯H/H⋯C, in descending order. The N⋯H/H⋯N contact percentage in (**I**) is notably lower than in (**II**), perhaps due to the presence of the ‘extra’ N atom in the pyrimidine ring in the latter. The percentage contributions of O⋯O and O⋯Cl contacts are close to zero in both structures, presumably reflecting the fact that ‘bare’ (unprotonated) O atoms and Cl^−^ anions avoid each other in the solid state for electrostatic reasons.

## 4. Database survey

A survey of the Cambridge Structural Database [CSD 2025.1 (released May 2025); Groom *et al.*, 2016 ▸] revealed 101 structures incorporating a pyridoxal (px) ring, of which 52 were protonated at the pyridine N atom, with a wide variety of substituents at the carbon atom (C1 in our numbering scheme) *trans* to the pyridine N atom. A total of 32 structures contain a pyroxidal ring–hydrazone grouping of which 16 are proton­ated at the pyridine N atom., while the px—CH=N—NH—C(=O)– atom connectivity is found in 18 structures (10 protonated, 8 unprotonated).

The structures of four hydro­chloride salts of pyroxidal–carbohydrazide–aromatic ring derivatives closely related to (**I**) and (**II**) include (using the nomenclature of the respective authors), ((3-hy­droxy-5-(hy­droxy­meth­yl)-2-methyl­pyridine-4-yl)methyl­ene)benzohydrazide hydro­chloride monohydrate (CSD refcode IGELOW; Back *et al.*, 2009 ▸), *N*-pyridoxyl­idene-*N*′-picolinoylhydrazine hydro­chloride monohydrate (PYPICZ; Domiano *et al.*, 1978 ▸), 3-hy­droxy-5-(hy­droxy­meth­yl)-2-methyl-4-((2-(pyridine-4-carbon­yl)hydrazinyl­idene)meth­yl)pyridin-1-ium chloride dimethyl sulfoxide solvate (VUYPOW; Mezey *et al.*, 2015 ▸) and 3-hy­droxy-5-(hy­droxy­meth­yl)-2-methyl-4-((2-(pyrimidine-2-carbon­yl)hydrazineyl­idene)meth­yl)pyridin-1-ium chloride monohydrate (XARHUX; Low, 2021 ▸).

**Figure 6:**
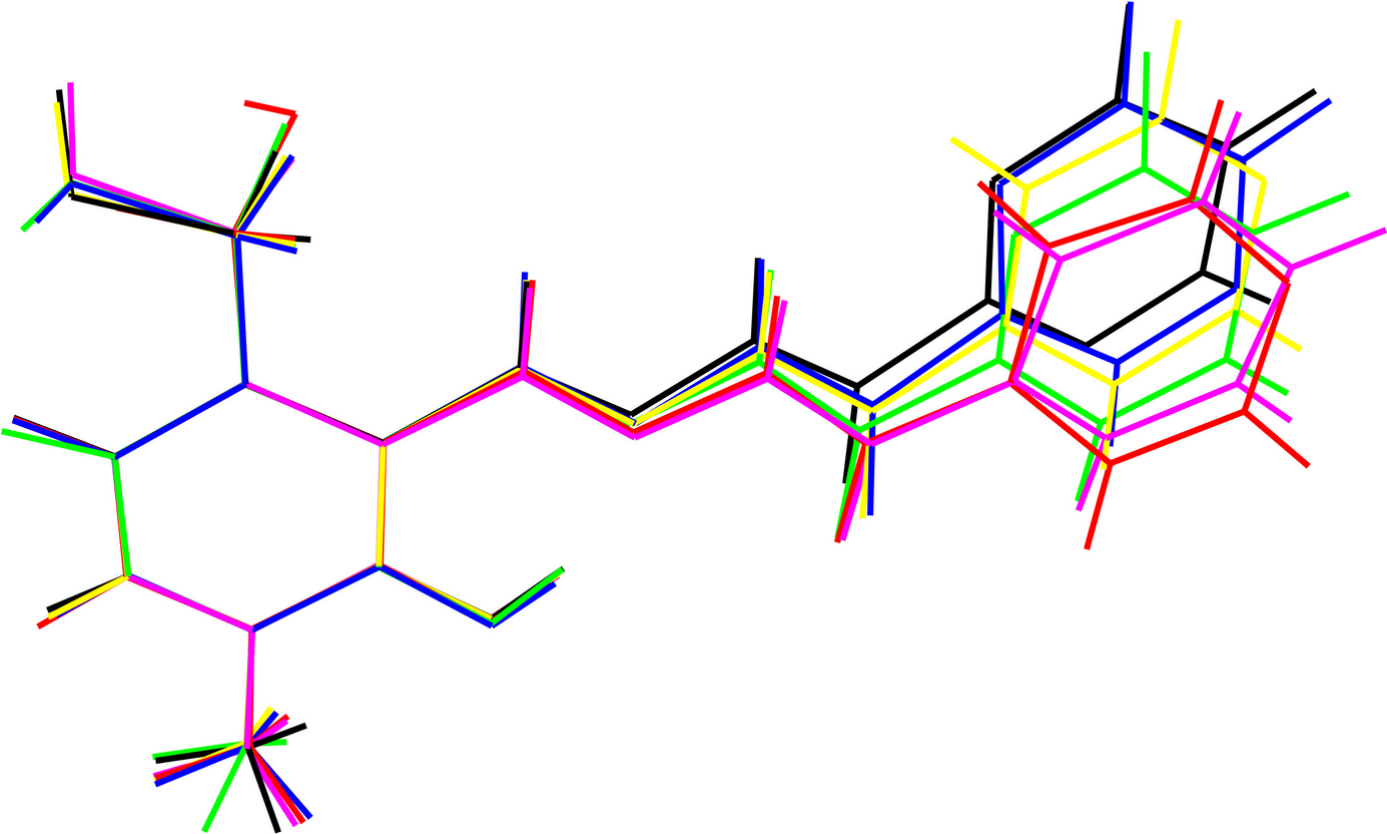
Overlay plot of the cations in (**I**) (red), (**II**) (blue), IGELOW (purple), PYPICZ (green), VUYPOW (yellow) and XARHUX (black). The conformations of the pyridoxal rings overlap almost perfectly.

Key structural data for (**I**), (**II**) and these phases are listed in Table 4 ▸. Despite their different hydrogen-bonding patterns, the cations in (**I**), (**II**) and the refcodes noted in the previous paragraph have similar conformations as shown in the overlay plot generated with QMOL (Gans & Shalloway, 2001 ▸) (Fig. 6 ▸). The ‘most different’ structure is (**I**), with a *gauche* disposition of the O atom of the hy­droxy­methyl group and a hydrogen bond to an N-atom acceptor from N1—H1n. All the other structures have an *anti* conformation for the O atom of the hy­droxy­methyl group and form an N1—H1*n*⋯Cl hydrogen bond.

## 5. Synthesis and crystallization

For the syntheses and spectroscopic data of (**I**) and (**II**), see Nogueira *et al.* (2019 ▸), where they were designated as compounds 2d and 2f, respectively. Single crystals [yellow plates for (**I**) and colourless slabs for (**II**)] were recrystallized from ethanol solution at room temperature.

## 6. Refinement

Crystal data, data collection and structure refinement details are summarized in Table 5 ▸. The N-and O-bound H atoms were located in difference maps and their positions were freely refined with *U*_iso_(H) = 1.2*U*_eq_(N, O). All the C-bound H atoms were located geometrically (C—H = 0.95–0.99 Å) and refined as riding atoms with *U*_iso_(H) = 1.2*U*_eq_(C) or 1.5*U*_eq_(methyl C). The methyl groups were allowed to rotate, but not to tip, to best fit the electron density. The crystal of (**I**) chosen for data collection was found to be twinned by 180° rotation about the [001] axis in reciprocal space or the [0.29,0.00,0.96] axis in direct space and was modelled as a non-merohedral two-component twin with a refined domain ratio of 0.4994 (11):0.5006 (11). Reflections were not merged (HKLF 5 card in SHELXL) and therefore *R*_Int_ for (**I**) is not defined. If the twinning was neglected and the reflections were merged (*R*_Int_ = 0.058), significantly poorer residuals (*R*1 = 0.046, *wR*2 = 0.132, *S* = 1.11) arose.

## Supplementary Material

Crystal structure: contains datablock(s) I, II, global. DOI: 10.1107/S2056989025007856/jy2065sup1.cif

Structure factors: contains datablock(s) I. DOI: 10.1107/S2056989025007856/jy2065Isup2.hkl

Structure factors: contains datablock(s) II. DOI: 10.1107/S2056989025007856/jy2065IIsup3.hkl

Supporting information file. DOI: 10.1107/S2056989025007856/jy2065Isup4.cml

Supporting information file. DOI: 10.1107/S2056989025007856/jy2065IIsup5.cml

CCDC references: 2485015, 2485014

Additional supporting information:  crystallographic information; 3D view; checkCIF report

## Figures and Tables

**Table 1:** Hydrogen-bond geometry (Å, °) for (**I**)

*D*—H⋯*A*	*D*—H	H⋯*A*	*D*⋯*A*	*D*—H⋯*A*
N1—H1*N*⋯N4^i^	0.99 (3)	1.82 (3)	2.809 (3)	175 (3)
N3—H2*N*⋯Cl1	0.85 (4)	2.26 (4)	3.103 (2)	170 (3)
O1—H1*O*⋯N2	0.74 (4)	1.89 (4)	2.553 (3)	148 (4)
O2—H2*O*⋯Cl1^ii^	0.81 (4)	2.28 (4)	3.081 (2)	168 (3)
O4—H1*W*⋯O3	0.97 (4)	2.08 (5)	3.027 (3)	163 (3)
O4—H2*W*⋯O5^iii^	0.90 (4)	1.87 (4)	2.751 (3)	166 (4)
O5—H3*W*⋯Cl1^iv^	0.90 (5)	2.37 (5)	3.247 (2)	166 (4)
O5—H4*W*⋯O4	0.95 (4)	1.84 (4)	2.758 (3)	162 (4)
C4—H4⋯O4^v^	0.95	2.42	3.347 (3)	166
C6—H6*A*⋯Cl1^vi^	0.98	2.82	3.495 (3)	127
C6—H6*C*⋯O3^iii^	0.98	2.72	3.380 (3)	125
C7—H7*B*⋯O2^iii^	0.99	2.59	3.530 (3)	159
C8—H8⋯Cl1	0.95	2.78	3.556 (2)	139
C11—H11⋯Cl1	0.95	2.64	3.555 (3)	162
C12—H12⋯O5^vii^	0.95	2.57	3.439 (3)	152
C13—H13⋯O2^viii^	0.95	2.45	3.351 (3)	159
C14—H14⋯Cl1^viii^	0.95	2.89	3.810 (3)	163

Symmetry codes: (i) 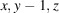; (ii) 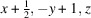; (iii) 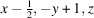; (iv) 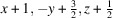; (v) 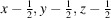; (vi) 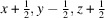; (vii) 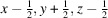; (viii) 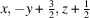.

**Table 2:** Hydrogen-bond geometry (Å, °) for (**II**)

*D*—H⋯*A*	*D*—H	H⋯*A*	*D*⋯*A*	*D*—H⋯*A*
N1—H1*N*⋯Cl1	0.80 (4)	2.29 (4)	3.089 (3)	174 (4)
N3—H2*N*⋯O4^i^	0.87 (4)	2.02 (4)	2.854 (4)	160 (3)
O1—H1*O*⋯N2	0.77 (4)	1.92 (4)	2.606 (3)	149 (4)
O2—H2*O*⋯Cl1^ii^	0.83 (4)	2.33 (4)	3.157 (3)	175 (4)
O4—H1*W*⋯Cl1^iii^	0.85 (4)	2.45 (4)	3.222 (3)	152 (4)
O4—H2*W*⋯O5	0.81 (4)	1.97 (4)	2.753 (4)	163 (4)
O5—H3*W*⋯O3	0.85 (5)	1.99 (5)	2.833 (3)	170 (4)
O5—H4*W*⋯O4^iv^	0.97 (4)	1.89 (4)	2.859 (4)	173 (4)
C4—H4⋯N4^v^	0.95	2.46	3.400 (4)	170
C8—H8⋯O4^i^	0.95	2.33	3.145 (4)	143
C11—H11⋯Cl1^vi^	0.95	2.84	3.724 (3)	155
C12—H12⋯O2^vi^	0.95	2.50	3.186 (4)	129

Symmetry codes: (i) 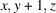; (ii) 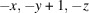; (iii) 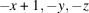; (iv) 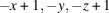; (v) 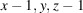; (vi) 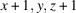.

**Table 3:** Hirshfeld fingerprint contact percentages for the cations in (**I**) and (**II**)

Contact type	(**I**)	(**II**)
H⋯H	39.2	36.3
O⋯H/H⋯O	19.9	16.7
C⋯H/H⋯C	12.6	9.0
N⋯H/H⋯N	6.0	11.6
H⋯Cl	6.5	7.5
C⋯C	4.5	6.0
C⋯O/O⋯C	0.7	4.2
C⋯N/N⋯C	9.1	4.8
O⋯O	0.3	0.0
O⋯Cl/Cl⋯O	0.0	0.0

**Table 4:** Key structural data for the cations in (**I**), (**II**) and related phases φ is the dihedral angle (°) between the pyridoxal ring and the pendant ring and ζ is the conformation of the C1—C5—C7—O2 grouping (our numbering scheme). The atom designations in the N1, N3 and O2 columns are the hydrogen-bond acceptors for these protonated atoms in the respective cations; N_p_ = pyridine, O_w_ = water; O_D_ = DMSO (di­methyl­sulfoxide). Atom O1 forms an intra­molecular O—H⋯N hydrogen bond in every case.

Compound	Space group	φ	ζ	N1	N3	O2
(**I**)	*Ia*	12.63 (12)	*gauche*	N_p_	Cl^−^	Cl^−^
(**II**)	*P* 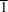	6.11 (15)	*anti*	Cl^−^	O_w_	Cl^−^
IGELOW	*Cc*	5.4	*anti*	Cl^−^	Cl^−^	O_w_
PYPICZ	*Cc*	5.1	*anti*	Cl^−^	Cl^−^	O_w_
VUYPOW	*P* 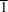	7.6	*anti*	Cl^−^	O_D_	Cl^−^
XARHUX	*P*2_1_/*c*	3.5	*anti*	Cl^−^	O_w_	Cl^−^

**Table 5:** Experimental details

	(**I**)	(**II**)
Crystal data		
Chemical formula	C_14_H_15_N_4_O_3_^+^·Cl^−^·2H_2_O	C_13_H_14_N_5_O_3_^+^·Cl^−^·2H_2_O
*M*_r_	358.78	359.77
Crystal system, space group	Monoclinic, *Ia*	Triclinic, *P* 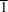
Temperature (K)	100	100
*a*, *b*, *c* (Å)	6.6920 (3), 14.0625 (5), 16.9446 (6)	7.5854 (2), 9.0709 (3), 11.4235 (3)
α, β, γ (°)	90, 96.821 (4), 90	87.954 (2), 89.397 (2), 86.604 (3)
*V* (Å^3^)	1583.31 (11)	784.10 (4)
*Z*	4	2
Radiation type	Mo *K*α	Mo *K*α
μ (mm^−1^)	0.28	0.28
Crystal size (mm)	0.15 × 0.05 × 0.02	0.04 × 0.04 × 0.01

Data collection		
Diffractometer	XtaLAB AFC12 (RCD3): Kappa single CCD	XtaLAB AFC12 (RCD3): Kappa single CCD
Absorption correction	Multi-scan (*CrysAlis PRO*; Rigaku OD, 2015 ▸)	Multi-scan (*CrysAlis PRO*; Rigaku OD, 2015 ▸)
*T*_min_, *T*_max_	0.817, 1.000	0.748, 1.000
No. of measured, independent and observed [*I* > 2σ(*I*)] reflections	3463, 3463, 3382	14113, 3586, 3448
*R*_int_	–	0.046
(sin θ/λ)_max_ (Å^−1^)	0.649	0.649

Refinement		
*R*[*F*^2^ > 2σ(*F*^2^)], *wR*(*F*^2^), *S*	0.028, 0.076, 1.04	0.088, 0.137, 1.43
No. of reflections	3463	3586
No. of parameters	243	242
No. of restraints	2	0
H-atom treatment	H atoms treated by a mixture of independent and constrained refinement	H atoms treated by a mixture of independent and constrained refinement
Δρ_max_, Δρ_min_ (e Å^−3^)	0.32, −0.17	0.48, −0.37
Absolute structure	Flack (1983 ▸)	–
Absolute structure parameter	0.43 (3)	–

Computer programs: *CrysAlis PRO* (Rigaku OD, 2015 ▸), *SHELXT* (Sheldrick, 2015*a* ▸), *SHELXL2019/2* (Sheldrick, 2015*b* ▸), *ORTEP-3 for Windows* (Farrugia, 2012 ▸) and *publCIF* (Westrip, 2010 ▸).

## supplementary crystallographic information

### (E)-3-Hydroxy-5-(hydroxymethyl)-2-methyl-4-{[(pyridin-4-ylformamido)imino]methyl}pyridin-1-ium chloride dihydrate (I) Crystal data

**Table:**

C_14_H_15_N_4_O_3_^+^·Cl^−^·2H_2_O	*F*(000) = 752
*M**_r_* = 358.78	*D*_x_ = 1.505 Mg m^−^^3^
Monoclinic, *I**a*	Mo *K*α radiation, λ = 0.71073 Å
*a* = 6.6920 (3) Å	Cell parameters from 8822 reflections
*b* = 14.0625 (5) Å	θ = 1.9–31.9°
*c* = 16.9446 (6) Å	µ = 0.28 mm^−^^1^
β = 96.821 (4)°	*T* = 100 K
*V* = 1583.31 (11) Å^3^	Plate, yellow
*Z* = 4	0.15 × 0.05 × 0.02 mm

### (E)-3-Hydroxy-5-(hydroxymethyl)-2-methyl-4-{[(pyridin-4-ylformamido)imino]methyl}pyridin-1-ium chloride dihydrate (I) Data collection

**Table:**

XtaLAB AFC12 (RCD3): Kappa single CCD diffractometer	3463 independent reflections
Radiation source: fine-focus sealed X-ray tube	3382 reflections with *I* > 2σ(*I*)
Graphite monochromator	θ_max_ = 27.5°, θ_min_ = 1.9°
ω scans	*h* = −8→8
Absorption correction: multi-scan (CrysAlisPro; Rigaku OD, 2015)	*k* = −18→18
*T*_min_ = 0.817, *T*_max_ = 1.000	*l* = −21→21
3463 measured reflections	

### (E)-3-Hydroxy-5-(hydroxymethyl)-2-methyl-4-{[(pyridin-4-ylformamido)imino]methyl}pyridin-1-ium chloride dihydrate (I) Refinement

**Table:**

Refinement on *F*^2^	Hydrogen site location: mixed
Least-squares matrix: full	H atoms treated by a mixture of independent and constrained refinement
*R*[*F*^2^ > 2σ(*F*^2^)] = 0.028	*w* = 1/[σ^2^(*F*_o_^2^) + (0.0538*P*)^2^ + 0.4094*P*] where *P* = (*F*_o_^2^ + 2*F*_c_^2^)/3
*wR*(*F*^2^) = 0.076	(Δ/σ)_max_ = 0.001
*S* = 1.04	Δρ_max_ = 0.32 e Å^−^^3^
3463 reflections	Δρ_min_ = −0.17 e Å^−^^3^
243 parameters	Absolute structure: Flack (1983)
2 restraints	Absolute structure parameter: 0.43 (3)
Primary atom site location: dual	

### (E)-3-Hydroxy-5-(hydroxymethyl)-2-methyl-4-{[(pyridin-4-ylformamido)imino]methyl}pyridin-1-ium chloride dihydrate (I) Special details

**Table:**

Geometry. All esds (except the esd in the dihedral angle between two l.s. planes) are estimated using the full covariance matrix. The cell esds are taken into account individually in the estimation of esds in distances, angles and torsion angles; correlations between esds in cell parameters are only used when they are defined by crystal symmetry. An approximate (isotropic) treatment of cell esds is used for estimating esds involving l.s. planes.
Refinement. Refined as a 2-component twin.

### (E)-3-Hydroxy-5-(hydroxymethyl)-2-methyl-4-{[(pyridin-4-ylformamido)imino]methyl}pyridin-1-ium chloride dihydrate (I) Fractional atomic coordinates and isotropic or equivalent isotropic displacement parameters (Å^2^)

**Table:**

	*x*	*y*	*z*	*U*_iso_*/*U*_eq_	
C1	0.1894 (4)	0.41717 (17)	0.41587 (15)	0.0089 (4)	
C2	0.2311 (4)	0.38525 (17)	0.49480 (15)	0.0102 (5)	
C3	0.2362 (4)	0.28718 (17)	0.51110 (16)	0.0107 (5)	
C4	0.1568 (4)	0.25379 (17)	0.37381 (14)	0.0113 (5)	
H4	0.131032	0.207330	0.333166	0.014*	
C5	0.1503 (4)	0.34915 (17)	0.35443 (14)	0.0098 (4)	
C6	0.2784 (4)	0.25102 (19)	0.59378 (16)	0.0155 (5)	
H6A	0.414244	0.270233	0.616103	0.023*	
H6B	0.268905	0.181482	0.593584	0.023*	
H6C	0.179980	0.277507	0.626196	0.023*	
C7	0.1043 (4)	0.37799 (16)	0.26815 (17)	0.0121 (5)	
H7A	0.074577	0.320447	0.235182	0.014*	
H7B	−0.016782	0.419004	0.261839	0.014*	
C8	0.1808 (4)	0.51891 (15)	0.39765 (14)	0.0092 (4)	
H8	0.152324	0.541553	0.344683	0.011*	
C9	0.2432 (4)	0.72744 (18)	0.51043 (15)	0.0103 (5)	
C10	0.2316 (3)	0.83284 (17)	0.49734 (15)	0.0102 (5)	
C11	0.2261 (4)	0.87597 (18)	0.42325 (16)	0.0133 (5)	
H11	0.226505	0.838917	0.376386	0.016*	
C12	0.2200 (4)	0.97495 (17)	0.41954 (16)	0.0133 (5)	
H12	0.218239	1.004509	0.369056	0.016*	
C13	0.2214 (4)	0.98771 (17)	0.55463 (15)	0.0120 (5)	
H13	0.218617	1.026386	0.600454	0.014*	
C14	0.2305 (4)	0.88987 (17)	0.56446 (15)	0.0110 (5)	
H14	0.235759	0.862334	0.615865	0.013*	
N1	0.1996 (3)	0.22622 (14)	0.45033 (13)	0.0106 (4)	
H1N	0.204 (5)	0.158 (2)	0.465 (2)	0.013*	
N2	0.2139 (3)	0.57609 (14)	0.45695 (14)	0.0109 (4)	
N3	0.2052 (3)	0.67197 (14)	0.44364 (13)	0.0101 (4)	
H2N	0.174 (5)	0.690 (2)	0.396 (2)	0.012*	
N4	0.2164 (3)	1.03054 (14)	0.48363 (13)	0.0124 (4)	
O1	0.2682 (3)	0.44138 (14)	0.55825 (11)	0.0157 (4)	
H1O	0.262 (5)	0.491 (3)	0.544 (2)	0.019*	
O2	0.2685 (3)	0.42794 (13)	0.24086 (11)	0.0150 (4)	
H2O	0.367 (6)	0.394 (3)	0.240 (2)	0.018*	
O3	0.2836 (3)	0.69357 (13)	0.57644 (11)	0.0164 (4)	
Cl1	0.12246 (8)	0.71352 (4)	0.26284 (4)	0.01433 (14)	
O4	0.4821 (3)	0.60393 (16)	0.72829 (14)	0.0276 (5)	
H1W	0.394 (6)	0.632 (3)	0.685 (3)	0.033*	
H2W	0.451 (6)	0.542 (3)	0.729 (2)	0.033*	
O5	0.8937 (3)	0.58262 (15)	0.75869 (14)	0.0254 (5)	
H3W	0.966 (6)	0.635 (3)	0.752 (2)	0.031*	
H4W	0.758 (6)	0.594 (3)	0.737 (2)	0.031*	

### (E)-3-Hydroxy-5-(hydroxymethyl)-2-methyl-4-{[(pyridin-4-ylformamido)imino]methyl}pyridin-1-ium chloride dihydrate (I) Atomic displacement parameters (Å^2^)

**Table:**

	*U*^11^	*U*^22^	*U*^33^	*U*^12^	*U*^13^	*U*^23^
C1	0.0098 (10)	0.0052 (10)	0.0117 (11)	−0.0003 (8)	0.0012 (8)	−0.0011 (8)
C2	0.0114 (10)	0.0079 (11)	0.0113 (11)	0.0000 (8)	0.0007 (9)	−0.0009 (9)
C3	0.0135 (12)	0.0083 (11)	0.0107 (12)	0.0011 (8)	0.0028 (9)	0.0009 (9)
C4	0.0140 (11)	0.0084 (10)	0.0122 (12)	0.0004 (9)	0.0045 (10)	−0.0020 (9)
C5	0.0113 (10)	0.0079 (10)	0.0103 (11)	0.0002 (8)	0.0021 (9)	−0.0013 (8)
C6	0.0252 (13)	0.0104 (11)	0.0106 (12)	0.0011 (10)	0.0010 (10)	0.0018 (10)
C7	0.0186 (12)	0.0084 (9)	0.0093 (11)	−0.0012 (9)	0.0017 (9)	0.0018 (10)
C8	0.0141 (10)	0.0038 (10)	0.0099 (11)	0.0008 (8)	0.0018 (9)	0.0012 (8)
C9	0.0139 (11)	0.0069 (11)	0.0104 (12)	0.0003 (9)	0.0026 (9)	0.0006 (9)
C10	0.0110 (10)	0.0059 (10)	0.0136 (12)	0.0000 (8)	0.0011 (8)	−0.0008 (8)
C11	0.0198 (13)	0.0078 (11)	0.0125 (12)	−0.0006 (9)	0.0028 (9)	−0.0007 (9)
C12	0.0196 (12)	0.0076 (11)	0.0124 (12)	−0.0011 (8)	0.0012 (9)	0.0016 (9)
C13	0.0167 (10)	0.0072 (11)	0.0124 (11)	−0.0003 (9)	0.0033 (9)	−0.0019 (9)
C14	0.0144 (11)	0.0093 (11)	0.0095 (11)	−0.0007 (8)	0.0027 (9)	−0.0004 (9)
N1	0.0130 (10)	0.0059 (9)	0.0129 (10)	0.0003 (7)	0.0023 (8)	0.0002 (8)
N2	0.0143 (10)	0.0052 (9)	0.0138 (10)	−0.0001 (7)	0.0034 (8)	0.0008 (8)
N3	0.0167 (10)	0.0034 (9)	0.0101 (10)	0.0006 (7)	0.0011 (8)	0.0001 (7)
N4	0.0158 (9)	0.0072 (9)	0.0140 (10)	−0.0006 (7)	0.0015 (8)	−0.0005 (8)
O1	0.0303 (11)	0.0062 (8)	0.0096 (9)	−0.0007 (7)	−0.0015 (8)	−0.0005 (7)
O2	0.0191 (9)	0.0117 (8)	0.0147 (9)	−0.0011 (7)	0.0049 (7)	0.0035 (7)
O3	0.0290 (10)	0.0093 (8)	0.0108 (9)	0.0013 (7)	0.0027 (7)	0.0012 (7)
Cl1	0.0198 (3)	0.0120 (2)	0.0109 (2)	0.0005 (2)	0.0003 (2)	0.0003 (2)
O4	0.0311 (12)	0.0185 (10)	0.0317 (13)	−0.0014 (9)	−0.0028 (10)	0.0009 (9)
O5	0.0311 (11)	0.0171 (10)	0.0270 (11)	−0.0022 (8)	−0.0011 (10)	0.0017 (9)

### (E)-3-Hydroxy-5-(hydroxymethyl)-2-methyl-4-{[(pyridin-4-ylformamido)imino]methyl}pyridin-1-ium chloride dihydrate (I) Geometric parameters (Å, º)

**Table:**

C1—C2	1.407 (3)	C9—C10	1.499 (3)
C1—C5	1.415 (3)	C10—C11	1.391 (3)
C1—C8	1.463 (3)	C10—C14	1.392 (3)
C2—O1	1.333 (3)	C11—C12	1.394 (3)
C2—C3	1.406 (3)	C11—H11	0.9500
C3—N1	1.340 (3)	C12—N4	1.341 (3)
C3—C6	1.486 (3)	C12—H12	0.9500
C4—N1	1.351 (3)	C13—N4	1.342 (3)
C4—C5	1.380 (3)	C13—C14	1.386 (3)
C4—H4	0.9500	C13—H13	0.9500
C5—C7	1.513 (3)	C14—H14	0.9500
C6—H6A	0.9800	N1—H1N	0.99 (3)
C6—H6B	0.9800	N2—N3	1.367 (3)
C6—H6C	0.9800	N3—H2N	0.85 (4)
C7—O2	1.427 (3)	O1—H1O	0.74 (4)
C7—H7A	0.9900	O2—H2O	0.81 (4)
C7—H7B	0.9900	O4—H1W	0.97 (4)
C8—N2	1.285 (3)	O4—H2W	0.90 (4)
C8—H8	0.9500	O5—H3W	0.90 (5)
C9—O3	1.216 (3)	O5—H4W	0.95 (4)
C9—N3	1.373 (3)		
			
C2—C1—C5	118.8 (2)	O3—C9—N3	122.3 (2)
C2—C1—C8	120.7 (2)	O3—C9—C10	121.7 (2)
C5—C1—C8	120.4 (2)	N3—C9—C10	116.0 (2)
O1—C2—C3	115.1 (2)	C11—C10—C14	119.0 (2)
O1—C2—C1	125.1 (2)	C11—C10—C9	124.0 (2)
C3—C2—C1	119.8 (2)	C14—C10—C9	117.0 (2)
N1—C3—C2	118.6 (2)	C10—C11—C12	118.3 (2)
N1—C3—C6	120.2 (2)	C10—C11—H11	120.9
C2—C3—C6	121.2 (2)	C12—C11—H11	120.9
N1—C4—C5	120.3 (2)	N4—C12—C11	123.3 (2)
N1—C4—H4	119.9	N4—C12—H12	118.3
C5—C4—H4	119.9	C11—C12—H12	118.3
C4—C5—C1	118.9 (2)	N4—C13—C14	123.3 (2)
C4—C5—C7	119.2 (2)	N4—C13—H13	118.3
C1—C5—C7	121.9 (2)	C14—C13—H13	118.3
C3—C6—H6A	109.5	C13—C14—C10	118.5 (2)
C3—C6—H6B	109.5	C13—C14—H14	120.7
H6A—C6—H6B	109.5	C10—C14—H14	120.7
C3—C6—H6C	109.5	C3—N1—C4	123.5 (2)
H6A—C6—H6C	109.5	C3—N1—H1N	116 (2)
H6B—C6—H6C	109.5	C4—N1—H1N	121 (2)
O2—C7—C5	111.7 (2)	C8—N2—N3	119.2 (2)
O2—C7—H7A	109.3	N2—N3—C9	115.1 (2)
C5—C7—H7A	109.3	N2—N3—H2N	117 (2)
O2—C7—H7B	109.3	C9—N3—H2N	128 (2)
C5—C7—H7B	109.3	C12—N4—C13	117.6 (2)
H7A—C7—H7B	107.9	C2—O1—H1O	107 (3)
N2—C8—C1	116.6 (2)	C7—O2—H2O	112 (3)
N2—C8—H8	121.7	H1W—O4—H2W	107 (4)
C1—C8—H8	121.7	H3W—O5—H4W	109 (3)
			
C5—C1—C2—O1	−179.2 (2)	N3—C9—C10—C11	−13.5 (3)
C8—C1—C2—O1	−0.9 (4)	O3—C9—C10—C14	−12.0 (4)
C5—C1—C2—C3	1.0 (4)	N3—C9—C10—C14	167.9 (2)
C8—C1—C2—C3	179.2 (2)	C14—C10—C11—C12	0.1 (3)
O1—C2—C3—N1	179.7 (2)	C9—C10—C11—C12	−178.5 (2)
C1—C2—C3—N1	−0.4 (4)	C10—C11—C12—N4	−0.9 (4)
O1—C2—C3—C6	0.6 (4)	N4—C13—C14—C10	−0.9 (4)
C1—C2—C3—C6	−179.6 (2)	C11—C10—C14—C13	0.8 (3)
N1—C4—C5—C1	0.1 (4)	C9—C10—C14—C13	179.5 (2)
N1—C4—C5—C7	179.3 (2)	C2—C3—N1—C4	−0.3 (4)
C2—C1—C5—C4	−0.8 (4)	C6—C3—N1—C4	178.9 (2)
C8—C1—C5—C4	−179.1 (2)	C5—C4—N1—C3	0.5 (4)
C2—C1—C5—C7	−180.0 (2)	C1—C8—N2—N3	−179.1 (2)
C8—C1—C5—C7	1.7 (4)	C8—N2—N3—C9	−179.8 (2)
C4—C5—C7—O2	−117.3 (2)	O3—C9—N3—N2	0.8 (4)
C1—C5—C7—O2	61.9 (3)	C10—C9—N3—N2	−179.15 (19)
C2—C1—C8—N2	0.2 (3)	C11—C12—N4—C13	0.8 (4)
C5—C1—C8—N2	178.5 (2)	C14—C13—N4—C12	0.2 (4)
O3—C9—C10—C11	166.6 (2)		

### (E)-3-Hydroxy-5-(hydroxymethyl)-2-methyl-4-{[(pyridin-4-ylformamido)imino]methyl}pyridin-1-ium chloride dihydrate (I) Hydrogen-bond geometry (Å, º)

**Table:**

*D*—H···*A*	*D*—H	H···*A*	*D*···*A*	*D*—H···*A*
N1—H1*N*···N4^i^	0.99 (3)	1.82 (3)	2.809 (3)	175 (3)
N3—H2*N*···Cl1	0.85 (4)	2.26 (4)	3.103 (2)	170 (3)
O1—H1*O*···N2	0.74 (4)	1.89 (4)	2.553 (3)	148 (4)
O2—H2*O*···Cl1^ii^	0.81 (4)	2.28 (4)	3.081 (2)	168 (3)
O4—H1*W*···O3	0.97 (4)	2.08 (5)	3.027 (3)	163 (3)
O4—H2*W*···O5^iii^	0.90 (4)	1.87 (4)	2.751 (3)	166 (4)
O5—H3*W*···Cl1^iv^	0.90 (5)	2.37 (5)	3.247 (2)	166 (4)
O5—H4*W*···O4	0.95 (4)	1.84 (4)	2.758 (3)	162 (4)
C4—H4···O4^v^	0.95	2.42	3.347 (3)	166
C6—H6*A*···Cl1^vi^	0.98	2.82	3.495 (3)	127
C6—H6*C*···O3^iii^	0.98	2.72	3.380 (3)	125
C7—H7*B*···O2^iii^	0.99	2.59	3.530 (3)	159
C8—H8···Cl1	0.95	2.78	3.556 (2)	139
C11—H11···Cl1	0.95	2.64	3.555 (3)	162
C12—H12···O5^vii^	0.95	2.57	3.439 (3)	152
C13—H13···O2^viii^	0.95	2.45	3.351 (3)	159
C14—H14···Cl1^viii^	0.95	2.89	3.810 (3)	163

Symmetry codes: (i) *x*, *y*−1, *z*; (ii) *x*+1/2, −*y*+1, *z*; (iii) *x*−1/2, −*y*+1, *z*; (iv) *x*+1, −*y*+3/2, *z*+1/2; (v) *x*−1/2, *y*−1/2, *z*−1/2; (vi) *x*+1/2, *y*−1/2, *z*+1/2; (vii) *x*−1/2, *y*+1/2, *z*−1/2; (viii) *x*, −*y*+3/2, *z*+1/2.

### (E)-3-Hydroxy-5-(hydroxymethyl)-2-methyl-4-{[(pyrimidin-2-ylformamido)imino]methyl}pyridin-1-ium chloride dihydrate (II) Crystal data

**Table:**

C_13_H_14_N_5_O_3_^+^·Cl^−^·2H_2_O	*Z* = 2
*M**_r_* = 359.77	*F*(000) = 376
Triclinic, *P*1	*D*_x_ = 1.524 Mg m^−^^3^
*a* = 7.5854 (2) Å	Mo *K*α radiation, λ = 0.71073 Å
*b* = 9.0709 (3) Å	Cell parameters from 4248 reflections
*c* = 11.4235 (3) Å	θ = 3.6–29.2°
α = 87.954 (2)°	µ = 0.28 mm^−^^1^
β = 89.397 (2)°	*T* = 100 K
γ = 86.604 (3)°	Slab, colourless
*V* = 784.10 (4) Å^3^	0.04 × 0.04 × 0.01 mm

### (E)-3-Hydroxy-5-(hydroxymethyl)-2-methyl-4-{[(pyrimidin-2-ylformamido)imino]methyl}pyridin-1-ium chloride dihydrate (II) Data collection

**Table:**

XtaLAB AFC12 (RCD3): Kappa single CCD diffractometer	3586 independent reflections
Radiation source: fine-focus sealed X-ray tube	3448 reflections with *I* > 2σ(*I*)
Graphite monochromator	*R*_int_ = 0.046
ω scans	θ_max_ = 27.5°, θ_min_ = 3.2°
Absorption correction: multi-scan (CrysAlisPro; Rigaku OD, 2015)	*h* = −9→9
*T*_min_ = 0.748, *T*_max_ = 1.000	*k* = −11→11
14113 measured reflections	*l* = −14→14

### (E)-3-Hydroxy-5-(hydroxymethyl)-2-methyl-4-{[(pyrimidin-2-ylformamido)imino]methyl}pyridin-1-ium chloride dihydrate (II) Refinement

**Table:**

Refinement on *F*^2^	Primary atom site location: dual
Least-squares matrix: full	Hydrogen site location: mixed
*R*[*F*^2^ > 2σ(*F*^2^)] = 0.088	H atoms treated by a mixture of independent and constrained refinement
*wR*(*F*^2^) = 0.137	*w* = 1/[σ^2^(*F*_o_^2^) + (0.0136*P*)^2^ + 1.6469*P*] where *P* = (*F*_o_^2^ + 2*F*_c_^2^)/3
*S* = 1.43	(Δ/σ)_max_ < 0.001
3586 reflections	Δρ_max_ = 0.48 e Å^−^^3^
242 parameters	Δρ_min_ = −0.36 e Å^−^^3^
0 restraints	

### (E)-3-Hydroxy-5-(hydroxymethyl)-2-methyl-4-{[(pyrimidin-2-ylformamido)imino]methyl}pyridin-1-ium chloride dihydrate (II) Special details

**Table:**

Geometry. All esds (except the esd in the dihedral angle between two l.s. planes) are estimated using the full covariance matrix. The cell esds are taken into account individually in the estimation of esds in distances, angles and torsion angles; correlations between esds in cell parameters are only used when they are defined by crystal symmetry. An approximate (isotropic) treatment of cell esds is used for estimating esds involving l.s. planes.

### (E)-3-Hydroxy-5-(hydroxymethyl)-2-methyl-4-{[(pyrimidin-2-ylformamido)imino]methyl}pyridin-1-ium chloride dihydrate (II) Fractional atomic coordinates and isotropic or equivalent isotropic displacement parameters (Å^2^)

**Table:**

	*x*	*y*	*z*	*U*_iso_*/*U*_eq_	
C1	0.4725 (4)	0.4993 (3)	0.2266 (3)	0.0079 (6)	
C2	0.4864 (4)	0.3444 (3)	0.2280 (3)	0.0079 (6)	
C3	0.4072 (4)	0.2679 (3)	0.1399 (3)	0.0086 (6)	
C4	0.2922 (4)	0.4970 (3)	0.0553 (3)	0.0084 (6)	
H4	0.222538	0.546413	−0.004305	0.010*	
C5	0.3692 (4)	0.5767 (3)	0.1391 (3)	0.0072 (6)	
C6	0.4226 (4)	0.1037 (3)	0.1359 (3)	0.0126 (7)	
H6A	0.391395	0.060692	0.212683	0.019*	
H6B	0.544209	0.071156	0.115600	0.019*	
H6C	0.342162	0.071263	0.076644	0.019*	
C7	0.3392 (4)	0.7439 (3)	0.1395 (3)	0.0088 (6)	
H7A	0.278121	0.772012	0.213111	0.011*	
H7B	0.454829	0.789112	0.137262	0.011*	
C8	0.5606 (4)	0.5814 (3)	0.3145 (3)	0.0094 (6)	
H8	0.555226	0.686354	0.310129	0.011*	
C9	0.8183 (4)	0.5209 (4)	0.5649 (3)	0.0093 (6)	
C10	0.9064 (4)	0.6210 (3)	0.6448 (3)	0.0083 (6)	
C11	0.9998 (4)	0.5607 (4)	0.7406 (3)	0.0098 (6)	
H11	1.010912	0.456382	0.752455	0.012*	
C12	1.0558 (4)	0.7921 (4)	0.7937 (3)	0.0128 (7)	
H12	1.105845	0.857195	0.845754	0.015*	
C13	0.9654 (4)	0.8515 (4)	0.6959 (3)	0.0118 (7)	
H13	0.958466	0.955603	0.682057	0.014*	
N1	0.3160 (4)	0.3485 (3)	0.0581 (2)	0.0089 (5)	
H1N	0.271 (5)	0.302 (4)	0.010 (3)	0.011*	
N2	0.6450 (3)	0.5108 (3)	0.3976 (2)	0.0090 (5)	
N3	0.7292 (4)	0.5939 (3)	0.4756 (2)	0.0095 (5)	
H2N	0.726 (5)	0.690 (4)	0.465 (3)	0.011*	
N4	1.0745 (4)	0.6464 (3)	0.8167 (2)	0.0120 (6)	
N5	0.8883 (4)	0.7665 (3)	0.6211 (2)	0.0106 (6)	
O1	0.5713 (3)	0.2600 (3)	0.3110 (2)	0.0118 (5)	
H1O	0.610 (5)	0.313 (4)	0.354 (3)	0.014*	
O2	0.2376 (3)	0.8000 (2)	0.04339 (19)	0.0121 (5)	
H2O	0.133 (5)	0.803 (4)	0.065 (3)	0.015*	
O3	0.8257 (3)	0.3867 (2)	0.58106 (19)	0.0124 (5)	
Cl1	0.15229 (10)	0.18625 (9)	−0.14370 (7)	0.01120 (19)	
O4	0.6679 (3)	−0.1073 (3)	0.3922 (2)	0.0171 (5)	
H1W	0.729 (5)	−0.097 (4)	0.330 (4)	0.020*	
H2W	0.698 (5)	−0.044 (5)	0.435 (4)	0.020*	
O5	0.7031 (4)	0.1000 (3)	0.5594 (2)	0.0207 (6)	
H3W	0.735 (6)	0.188 (5)	0.557 (4)	0.025*	
H4W	0.579 (6)	0.107 (4)	0.581 (4)	0.025*	

### (E)-3-Hydroxy-5-(hydroxymethyl)-2-methyl-4-{[(pyrimidin-2-ylformamido)imino]methyl}pyridin-1-ium chloride dihydrate (II) Atomic displacement parameters (Å^2^)

**Table:**

	*U*^11^	*U*^22^	*U*^33^	*U*^12^	*U*^13^	*U*^23^
C1	0.0083 (15)	0.0092 (15)	0.0067 (14)	−0.0016 (12)	0.0012 (12)	−0.0031 (11)
C2	0.0080 (14)	0.0102 (15)	0.0055 (14)	0.0001 (12)	0.0011 (11)	−0.0001 (11)
C3	0.0070 (14)	0.0104 (15)	0.0084 (14)	−0.0005 (12)	0.0017 (12)	0.0001 (12)
C4	0.0087 (14)	0.0093 (15)	0.0077 (14)	−0.0035 (12)	−0.0012 (11)	−0.0008 (11)
C5	0.0079 (14)	0.0076 (14)	0.0061 (14)	0.0003 (12)	0.0029 (11)	0.0006 (11)
C6	0.0166 (17)	0.0093 (16)	0.0120 (16)	−0.0007 (13)	−0.0019 (13)	−0.0030 (12)
C7	0.0125 (15)	0.0062 (14)	0.0081 (14)	−0.0027 (12)	−0.0019 (12)	−0.0012 (11)
C8	0.0085 (15)	0.0099 (15)	0.0104 (15)	−0.0032 (12)	−0.0002 (12)	−0.0032 (12)
C9	0.0054 (14)	0.0150 (16)	0.0076 (14)	−0.0012 (12)	0.0014 (12)	−0.0026 (12)
C10	0.0087 (15)	0.0101 (15)	0.0064 (14)	−0.0012 (12)	0.0014 (11)	−0.0022 (11)
C11	0.0099 (15)	0.0106 (15)	0.0092 (15)	−0.0022 (12)	0.0005 (12)	−0.0038 (12)
C12	0.0111 (16)	0.0159 (17)	0.0122 (15)	−0.0055 (13)	0.0006 (13)	−0.0069 (13)
C13	0.0156 (16)	0.0088 (15)	0.0116 (15)	−0.0043 (13)	−0.0005 (13)	−0.0015 (12)
N1	0.0108 (13)	0.0103 (13)	0.0061 (12)	−0.0022 (10)	−0.0020 (10)	−0.0034 (10)
N2	0.0083 (13)	0.0116 (13)	0.0076 (12)	−0.0021 (10)	−0.0031 (10)	−0.0037 (10)
N3	0.0135 (14)	0.0095 (13)	0.0061 (12)	−0.0028 (11)	−0.0028 (10)	−0.0022 (10)
N4	0.0126 (14)	0.0159 (14)	0.0079 (13)	−0.0037 (11)	−0.0010 (11)	−0.0008 (11)
N5	0.0117 (13)	0.0120 (13)	0.0085 (13)	−0.0027 (11)	−0.0017 (10)	−0.0029 (10)
O1	0.0155 (12)	0.0098 (11)	0.0102 (11)	−0.0011 (9)	−0.0066 (9)	−0.0011 (9)
O2	0.0138 (12)	0.0134 (12)	0.0089 (11)	0.0005 (10)	−0.0052 (9)	0.0015 (9)
O3	0.0160 (12)	0.0097 (11)	0.0118 (11)	−0.0019 (9)	−0.0028 (9)	−0.0025 (9)
Cl1	0.0123 (4)	0.0120 (4)	0.0097 (3)	−0.0017 (3)	−0.0035 (3)	−0.0036 (3)
O4	0.0243 (14)	0.0148 (13)	0.0130 (12)	−0.0059 (11)	−0.0032 (11)	−0.0040 (10)
O5	0.0259 (15)	0.0126 (13)	0.0245 (14)	−0.0039 (11)	−0.0045 (11)	−0.0067 (10)

### (E)-3-Hydroxy-5-(hydroxymethyl)-2-methyl-4-{[(pyrimidin-2-ylformamido)imino]methyl}pyridin-1-ium chloride dihydrate (II) Geometric parameters (Å, º)

**Table:**

C1—C2	1.402 (4)	C9—N3	1.360 (4)
C1—C5	1.417 (4)	C9—C10	1.500 (4)
C1—C8	1.461 (4)	C10—N5	1.337 (4)
C2—O1	1.343 (4)	C10—C11	1.388 (4)
C2—C3	1.403 (4)	C11—N4	1.339 (4)
C3—N1	1.339 (4)	C11—H11	0.9500
C3—C6	1.489 (4)	C12—N4	1.338 (4)
C4—N1	1.347 (4)	C12—C13	1.392 (5)
C4—C5	1.375 (4)	C12—H12	0.9500
C4—H4	0.9500	C13—N5	1.334 (4)
C5—C7	1.520 (4)	C13—H13	0.9500
C6—H6A	0.9800	N1—H1N	0.80 (4)
C6—H6B	0.9800	N2—N3	1.373 (4)
C6—H6C	0.9800	N3—H2N	0.87 (4)
C7—O2	1.410 (4)	O1—H1O	0.77 (4)
C7—H7A	0.9900	O2—H2O	0.83 (4)
C7—H7B	0.9900	O4—H1W	0.85 (4)
C8—N2	1.282 (4)	O4—H2W	0.81 (4)
C8—H8	0.9500	O5—H3W	0.85 (5)
C9—O3	1.223 (4)	O5—H4W	0.97 (4)
			
C2—C1—C5	119.1 (3)	C1—C8—H8	120.2
C2—C1—C8	121.1 (3)	O3—C9—N3	124.2 (3)
C5—C1—C8	119.8 (3)	O3—C9—C10	122.2 (3)
O1—C2—C1	124.1 (3)	N3—C9—C10	113.6 (3)
O1—C2—C3	115.7 (3)	N5—C10—C11	122.7 (3)
C1—C2—C3	120.2 (3)	N5—C10—C9	117.8 (3)
N1—C3—C2	117.3 (3)	C11—C10—C9	119.5 (3)
N1—C3—C6	120.5 (3)	N4—C11—C10	121.4 (3)
C2—C3—C6	122.2 (3)	N4—C11—H11	119.3
N1—C4—C5	119.8 (3)	C10—C11—H11	119.3
N1—C4—H4	120.1	N4—C12—C13	122.1 (3)
C5—C4—H4	120.1	N4—C12—H12	118.9
C4—C5—C1	118.6 (3)	C13—C12—H12	118.9
C4—C5—C7	120.5 (3)	N5—C13—C12	121.9 (3)
C1—C5—C7	120.9 (3)	N5—C13—H13	119.1
C3—C6—H6A	109.5	C12—C13—H13	119.1
C3—C6—H6B	109.5	C3—N1—C4	124.9 (3)
H6A—C6—H6B	109.5	C3—N1—H1N	115 (3)
C3—C6—H6C	109.5	C4—N1—H1N	120 (3)
H6A—C6—H6C	109.5	C8—N2—N3	116.8 (3)
H6B—C6—H6C	109.5	C9—N3—N2	117.6 (3)
O2—C7—C5	112.2 (2)	C9—N3—H2N	123 (2)
O2—C7—H7A	109.2	N2—N3—H2N	120 (2)
C5—C7—H7A	109.2	C12—N4—C11	116.1 (3)
O2—C7—H7B	109.2	C13—N5—C10	115.8 (3)
C5—C7—H7B	109.2	C2—O1—H1O	107 (3)
H7A—C7—H7B	107.9	C7—O2—H2O	107 (3)
N2—C8—C1	119.5 (3)	H1W—O4—H2W	105 (4)
N2—C8—H8	120.2	H3W—O5—H4W	106 (4)
			
C5—C1—C2—O1	−175.6 (3)	N3—C9—C10—N5	0.3 (4)
C8—C1—C2—O1	3.5 (5)	O3—C9—C10—C11	0.0 (5)
C5—C1—C2—C3	3.6 (4)	N3—C9—C10—C11	179.1 (3)
C8—C1—C2—C3	−177.2 (3)	N5—C10—C11—N4	1.4 (5)
O1—C2—C3—N1	177.6 (3)	C9—C10—C11—N4	−177.3 (3)
C1—C2—C3—N1	−1.7 (4)	N4—C12—C13—N5	1.8 (5)
O1—C2—C3—C6	−2.4 (4)	C2—C3—N1—C4	−1.2 (5)
C1—C2—C3—C6	178.3 (3)	C6—C3—N1—C4	178.9 (3)
N1—C4—C5—C1	0.1 (4)	C5—C4—N1—C3	2.0 (5)
N1—C4—C5—C7	−178.1 (3)	C1—C8—N2—N3	178.2 (3)
C2—C1—C5—C4	−2.8 (4)	O3—C9—N3—N2	−1.2 (5)
C8—C1—C5—C4	178.0 (3)	C10—C9—N3—N2	179.7 (2)
C2—C1—C5—C7	175.4 (3)	C8—N2—N3—C9	179.5 (3)
C8—C1—C5—C7	−3.7 (4)	C13—C12—N4—C11	−0.6 (5)
C4—C5—C7—O2	−3.6 (4)	C10—C11—N4—C12	−1.0 (4)
C1—C5—C7—O2	178.2 (3)	C12—C13—N5—C10	−1.3 (5)
C2—C1—C8—N2	−3.3 (5)	C11—C10—N5—C13	−0.3 (4)
C5—C1—C8—N2	175.8 (3)	C9—C10—N5—C13	178.5 (3)
O3—C9—C10—N5	−178.9 (3)		

### (E)-3-Hydroxy-5-(hydroxymethyl)-2-methyl-4-{[(pyrimidin-2-ylformamido)imino]methyl}pyridin-1-ium chloride dihydrate (II) Hydrogen-bond geometry (Å, º)

**Table:**

*D*—H···*A*	*D*—H	H···*A*	*D*···*A*	*D*—H···*A*
N1—H1*N*···Cl1	0.80 (4)	2.29 (4)	3.089 (3)	174 (4)
N3—H2*N*···O4^i^	0.87 (4)	2.02 (4)	2.854 (4)	160 (3)
O1—H1*O*···N2	0.77 (4)	1.92 (4)	2.606 (3)	149 (4)
O2—H2*O*···Cl1^ii^	0.83 (4)	2.33 (4)	3.157 (3)	175 (4)
O4—H1*W*···Cl1^iii^	0.85 (4)	2.45 (4)	3.222 (3)	152 (4)
O4—H2*W*···O5	0.81 (4)	1.97 (4)	2.753 (4)	163 (4)
O5—H3*W*···O3	0.85 (5)	1.99 (5)	2.833 (3)	170 (4)
O5—H4*W*···O4^iv^	0.97 (4)	1.89 (4)	2.859 (4)	173 (4)
C4—H4···N4^v^	0.95	2.46	3.400 (4)	170
C8—H8···O4^i^	0.95	2.33	3.145 (4)	143
C11—H11···Cl1^vi^	0.95	2.84	3.724 (3)	155
C12—H12···O2^vi^	0.95	2.50	3.186 (4)	129

Symmetry codes: (i) *x*, *y*+1, *z*; (ii) −*x*, −*y*+1, −*z*; (iii) −*x*+1, −*y*, −*z*; (iv) −*x*+1, −*y*, −*z*+1; (v) *x*−1, *y*, *z*−1; (vi) *x*+1, *y*, *z*+1.
